# Carbon Dioxide Absorption and Release Properties of Pyrolysis Products of Dolomite Calcined in Vacuum Atmosphere

**DOI:** 10.1155/2014/862762

**Published:** 2014-07-16

**Authors:** Fei Wang, Toshihiro Kuzuya, Shinji Hirai, Jihua Li, Te Li

**Affiliations:** ^1^Key Laboratory of Tropical Crop Products Processing of Ministry of Agriculture, Agriculture Products Processing Research Institute, Chinese Academy of Tropical Agricultural Sciences, Zhanjiang 524001, China; ^2^Division of Engineering for Composite Function, Muroran Institute of Technology, 27-1 Mizumoto-cho, Muroran, Hokkaido 050-8585, Japan

## Abstract

The decomposition of dolomite into CaO and MgO was performed at 1073 K in vacuum and at 1273 K in an Ar atmosphere. The dolomite calcined in vacuum was found to have a higher specific surface area and a higher micropore volume when compared to the dolomite calcined in the Ar atmosphere. These pyrolysis products of dolomite were reacted with CO_2_ at 673 K for 21.6 ks. On the absorption of CO_2_, the formation of CaCO_3_ was observed. The degree of absorption of the dolomite calcined in vacuum was determined to be above 50%, which was higher than the degree of absorption of the dolomite calcined in the Ar atmosphere. The CO_2_ absorption and release procedures were repeated three times for the dolomite calcined in vacuum. The specific surface area and micropore volume of calcined dolomite decreased with successive repetitions of the CO_2_ absorption and release cycles leading to a decrease in the degree of absorption of CO_2_.

## 1. Introduction

Mixed powders of CaO and MgO obtained by the pyrolysis of dolomite ore are considered as promising materials for the absorption of CO_2_ [1] because of their porous structure, reusability, and low cost [2]. The mixed powders react reversibly with CO_2_, as shown in (1) below:

(1) $$\begin{array}{c} \text{CaO}\left(\text{s}\right)+\text{MgO}\left(\text{s}\right)+2{\text{CO}}_{2}\left(\text{g}\right)=\text{CaMg}{\left({\text{CO}}_{3}\right)}_{2} \end{array}$$

A typical sequestration process of CO_2_ by a sorbent involves the use of two circulating fluidized beds. One is operated in the temperature range 673–973 K as a carbonator, and the other is operated in the temperature range 1023–1223 K as a cracker [2]. In theory, the volume of CO_2_ absorbed by the mixed powders from room temperature to 1100 K is 800–1600 times larger than the volume absorbed by pure CaO and MgO powders; the absorbed CO_2_ can be released when the powders are heated above 1100 K. Therefore, dolomite can be used for the selective absorption of CO_2_ from gas mixtures of H_2_ and CO_2_ such as those evolved from a water-gas shift reaction (2) or the decomposition of dimethyl ether (DME). Consider the following:

(2) $$\begin{array}{c} \text{CO}+{\text{H}}_{2}\text{O}={\text{CO}}_{2}+{\text{H}}_{2} \end{array}$$

There have been several reports on the properties of dolomite as a CO_2_ absorbent. The absorption performance of the pyrolysis products of dolomite depends on a number of factors such as the calcination conditions, CO_2_ flow rate, absorption temperature, and atmosphere. Among these factors, the calcination conditions strongly affect the absorption performance, because the porous structure of dolomite, which facilitates the diffusion of CO_2_, is formed and refined during the decomposition procedure [3, 4]. Kristóf-Makó and Juhász [5] indicated that the decomposition of dolomite was divided into two steps, which was restrained by the reaction temperature and atmosphere; Beruto et al. (2002) reported that the dolomite was decomposed at high CO_2_ pressure in the temperature range of 913–973 K. And they found that the kinetic analysis of the TG curves yields a total apparent enthalpy for the decomposition equal to 440 ± 10 kJ*·*mol^−1^ for the partial decomposition to the calcite and MgO. Rodriguez-Navarro et al. [6] reported the MgCO_3_ pseudophase in dolomite was first decomposed, leaving the undecomposed CaCO_3_ pseudophase at first step in air or in a CO_2_ atmosphere. On the analysis of the kinetics of partial decomposition of dolomite as a function of the CO_2_ pressure, Galai et al. [7] found that the rate-limiting step in the growth of MgO and CaCO_3_ can be controlled by the diffusion of magnesium through MgO phase surface. Gallucci et al. [8] introduced a mathematical model for the absorption of CO_2_ by the pyrolysis products of dolomite, based on the assumption that the solid particles of dolomite are made up of very small spherical grains. For the absorption process, which can be described as the carbonation of CaO–MgO mixture, the authors found good agreement between the model predictions and experimental data, as testified by the scanning electron microscopy (SEM) images of dolomite and the thermogravimetry (TG) curves of CO_2_ absorption process.

Stendardo and Foscolo [9] discussed a mathematical model of a heterogeneous gas-solid phase reaction taking place within very small spherical grains of the particles of a reactant. Their model was able to encapsulate changes in the structure of the spherical grains, by the introduction of a variable diffusion coefficient of the gaseous reactant. Beruto et al. [10, 11] found that a humid atmosphere causes the pyrolysis products of dolomite to show a high activity for CO_2_ absorption. They proved that though H_2_O(g) cannot change the properties of the solid products formed out of MgO, CaCO_3_, and solid solutions of MgO in CaCO_3_, it can increase the growth rate of the MgO particles. During hydration, CaO particles are not stable and they transform to Ca(OH)_2_ (3) and (4). Ewing et al. [12] introduced the pressure of CO_2_ which could influence the change of specific surface area of CaO. [13] And sintering of the CaO is negligible at 650°C in CO_2_ pressures of ≤0.1 torr, but sintering is pronounced in CO_2_, at 9 torr pressure. Beruto and Searcy [14] reported that the reactive crystalline material was synthesized during incomplete decomposition of calcite in vacuum atmosphere, with three-layer structure. And the layer of a poorly crystalline material was present between the undecomposed CaCO_3_ and a layer of normal polycrystalline CaO. Consider

(3) $$\begin{array}{c} \text{CaO}\left(\text{s}\right)+{\text{H}}_{2}\text{O}\left(\text{g}\right)=\text{Ca}{\left(\text{OH}\right)}_{2}\left(\text{s}\right) \end{array}$$

(4) Ca(OH)2(s)+CO2(g)=CaCO3(s)+H2O(g)

A lower decomposition temperature has a positive influence on the CO_2_ absorption properties of Ca-based absorbents, because particle agglomeration is minimized, leading to the preservation of their highly porous structure [3]. According to the theory of reaction rates, a decrease in the concentration of CO_2_ in the reactants can lower the decomposition temperature of dolomite. Since previous reports on dolomite had not focused on this aspect, we employed vacuum conditions for the calcination of dolomite and studied the corresponding effects on the absorption of CO_2_.

In this study, the CO_2_ absorption and release properties of the pyrolysis products of dolomite calcined in an Ar atmosphere and under vacuum were evaluated using TG. In addition, changes in their specific surface area and micropore volume were also investigated in a cyclic absorption and release procedure.

## 2. Experimental Procedure

Crystallized dolomite powder (Yoshizawa Lime Industry Co., Ltd., Japan) with an average particle diameter of 45 *μ*m and a specific surface area of 4.76 m^2^ *·*g^−1^ was used to prepare the CO_2_ absorbents. The chemical components of dolomite are listed in Table 1.

The CO_2_ absorption and release properties were evaluated by thermogravimetric analysis (TGA) and differential thermal analysis (DTA) performed using a thermogravimetry instrument (TG: TG-DTA2000S, MAC Science CO., Ltd., Kanagawa, Japan). For sample preparation, dolomite was calcined in an Ar atmosphere at 1273 K and separately in vacuum at 1073 K in the TG apparatus. The heating rate was 10 K*·*min^−1^, and the flow rate of Ar was 100 mL*·*min^−1^. After calcination, the pyrolysis products of dolomite were allowed to cool to room temperature in an Ar atmosphere. For absorption tests, the samples were heated up to 673 K in an Ar atmosphere with a heating rate of 10 K*·*min^−1^ and were kept at this temperature for 21.6 ks in a CO_2_ atmosphere. The flow rate of CO_2_ was 300 mL*·*min^−1^. For cyclic tests, the release and absorption steps were repeated three times under the same conditions.

The structure of the samples was studied with an X-ray diffractometer (Rint-UltIma+, Rigaku Corp., Tokyo, Japan) having a monochromatic CuK*α* radiation as the source. The specific surface area and micropore volume of the samples were measured using the Brunauer-Emmett-Teller (BET) five-point method under 77 K in N_2_ atmosphere (Autosorb-1, Quantachrome, FL, USA). SEM and energy-dispersive X-ray spectroscopy (EDS) analysis of the samples were performed using a SEM apparatus (JSM-6610LV, JEOL, Tokyo, Japan).

The degree of absorption was determined using the following equation under the assumption that the CO_2_ absorption reaction proceeds according to (1). The CO_2_ absorption per gram of CO_2_-saturated samples was calculated from (6) and was estimated to be 0.8702 g:

(5) WCO2=(WCaOF.W.CaO+WMgOF.W.MgO)×F.W.CO2=0.4666 g

(6) Ws=WCO2Wdolomits−WLoss=0.8702,

where *W*_CO_2__ is the stoichiometric absorption weight of CO_2_ for pure dolomite and *W*_CaO_, *W*_MgO_, and Wdolomite are the weights of CaO, MgO, and calcined dolomite, respectively. F.W._CaO_, F.W._MgO_, and F.W._CO_2__ are the molecular weights (g*·*mol^−1^) of CaO, MgO, and CO_2_, respectively. *W*_*S*_ is the theoretical maximum degree of absorption of CO_2_ and Wloss is the loss of fusion, as shown in Table 1. The actual degree of absorption was calculated from the following equation:

(7) $$\begin{array}{c} A_{N}=\frac{\Delta W}{\left(W_{S}\times W_{N-1}\right)}, \end{array}$$

where *A*_*N*_ represents the actual cyclic degree of absorption of CO_2_, *N* is the number of absorption cycles, Δ*W* is the change in weight during the absorption process, and *W*_*N*−1_ is the weight of the sample after calcination.

## 3. Results and Discussion 

### 3.1. XRD Analyses of Dolomite and Its Pyrolysis Products

**Figure 1:**
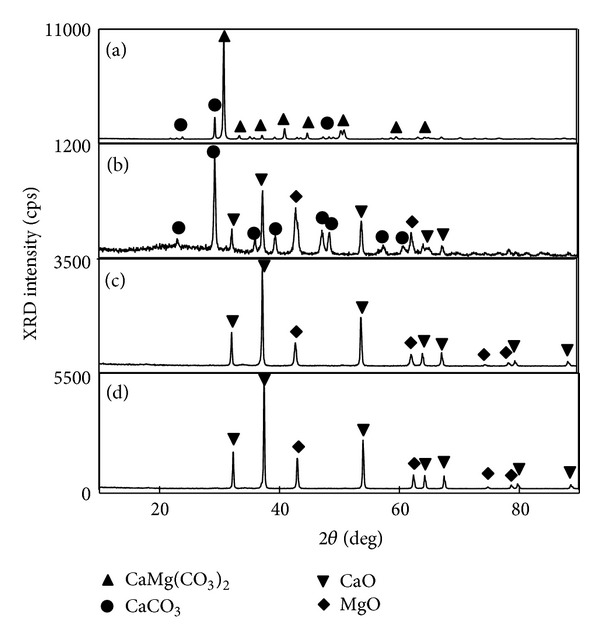
XRD patterns of the dolomite raw material and its pyrolysis products fabricated under different conditions. (a) Raw material, (b) pyrolysis products calcinated in an Ar atmosphere at 1173 K for 3.6 ks, (c) pyrolysis products calcinated in an Ar atmosphere at 1273 K for 3.6 ks, and (d) pyrolysis products calcinated in vacuum at 1073 K for 3.6 ks.

The X-ray diffraction (XRD) patterns of the dolomite raw material and its pyrolysis products fabricated under different conditions are shown in Figure 1. Figure 1(a) shows that calcium magnesium carbonate (CaMg (CO_3_)_2_) and CaCO_3_ were found in the dolomite raw material. The dolomite sample calcined in an Ar atmosphere at 1173 K contained a mixture of CaCO_3_, CaO, and MgO, as shown in Figure 1(b). However, when the calcination temperature was raised to 1273 K, the characteristic peaks of CaCO_3_ disappeared possibly due to its decomposition into CaO and CO_2_ (Figure 1(c)). Figure 1(d) shows the effect of a reduced decomposition temperature on the structure of the dolomite raw material.

### 3.2. Thermal Decomposition of Dolomite

**Figure 2:**
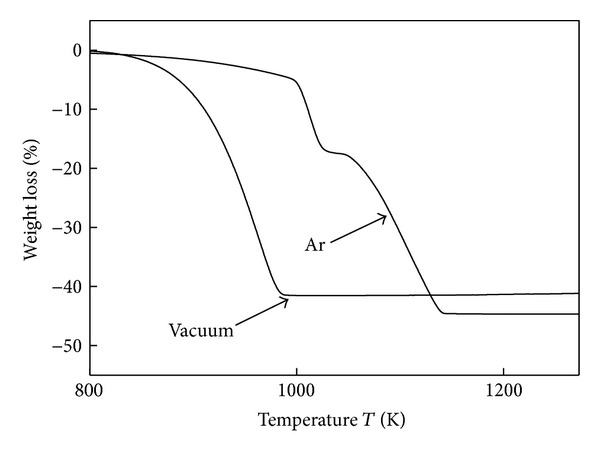
TG curves of dolomite decomposition in an Ar atmosphere and vacuum.

The TG results shown in Figure 2 corroborate well with the results of the XRD analyses. The TG curves of dolomite samples calcined under different conditions showed a weight loss of approximately 45%, which corresponds to the estimated weight loss of dolomite when it is completely decomposed into three components ((9) and (10)). For the dolomite sample calcined in vacuum, decomposition started at 800 K, which was lower than the decomposition temperature of dolomite calcined in an Ar atmosphere. As reported from Silcox et al. [15], the relationship between calcination temperature and equilibrium decomposition pressure is shown as (8). Calcium carbonate will be decomposed when its decomposition pressure above the carbon dioxide pressure around the particles; so in vacuum atmosphere, inert decomposition pressure is always higher than external carbon dioxide pressure, and the reaction will happen in relative lower temperature. Moreover,

(8) Peq=4.137×107exp⁡(−20474T)atm.

Previous studies have reported that dolomite decomposes in two steps at a high partial pressure of CO_2_. In the first step, CaCO_3_, MgO, and CO_2_ are formed, as shown in (9), and subsequently, CaCO_3_ also decomposes into CaO and CO_2_ (10) [5, 10, 11]

(9) CaCO3·MgCO3=CaCO3(s) + MgO(s)+CO2(g)(973−1073 K)

(10) CaCO3=CaO(s)+CO2(g)(>1173 K)

**Figure 3:**
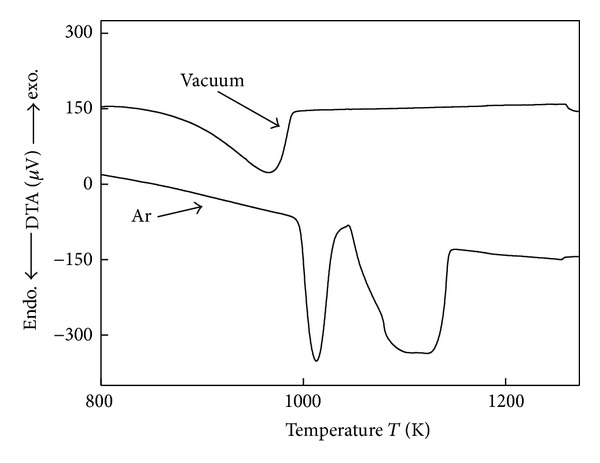
DTA curves of dolomite decomposition in an Ar atmosphere and vacuum.

**Figure 4:**
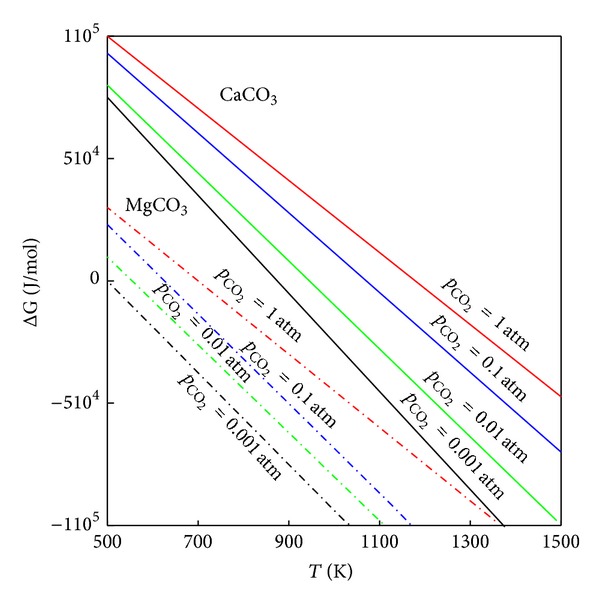
The Gibbs free energy changes of the decomposition reaction of CaCO_3_ and MgCO_3_ at *p*_CO_2__ = 0.001 ~ 1 atm.

For the calcination of dolomite in Ar and vacuum atmosphere, the DTA results are shown in Figure 3. The two endothermic peaks (A) and (B) can be attributed to the enthalpy changes of the decomposition reactions (9) and (10), respectively. The Gibbs free energy change of the decomposition reaction of dolomite can be represented by the following equations:

(11) $$\begin{array}{c} \Delta \text{G}=\Delta G_{0}+RT\times \ln\{\frac{\left(a_{\text{CaC}{\text{O}}_{3}}\times a_{\text{MgO}}\times p_{\text{C}{\text{O}}_{2}}\right)}{a_{\text{CaMg}{\left(\text{C}{\text{O}}_{3}\right)}_{2}}}\} \end{array}$$

(12) $$\begin{array}{c} \Delta \text{G}=\Delta G_{0}+RT\times \ln\{\frac{\left(a_{\text{CaO}}\times a_{\text{MgO}}\times p_{\text{C}{\text{O}}_{2}}\right)}{a_{\text{CaMg}{\left(\text{C}{\text{O}}_{3}\right)}_{2}}}\}, \end{array}$$

where Δ*G*, Δ*G*°, *T*, *a*, and *p* represent the Gibbs free energy change (kJ*·*mol^−1^), standard free energy change (kJ*·*mol^−1^), temperature (K), activity, and partial pressure, respectively. The activities of CaCO_3_, MgCO_3_, MgO, and CaO were all assumed to be 1.  Figure 4 illustrates the dependence of the decomposition temperature of dolomite on the partial pressure of CO_2_ for reactions in (9) and (10), which was determined from the thermodynamic data collected by Barin et al. [16, 17]. The decomposition temperature decreased with the decrease in the partial pressure of CO_2_ (*p*_CO_2__). This trend can also be seen in the results shown in Figures 2 and 3.

**Figure 5:**
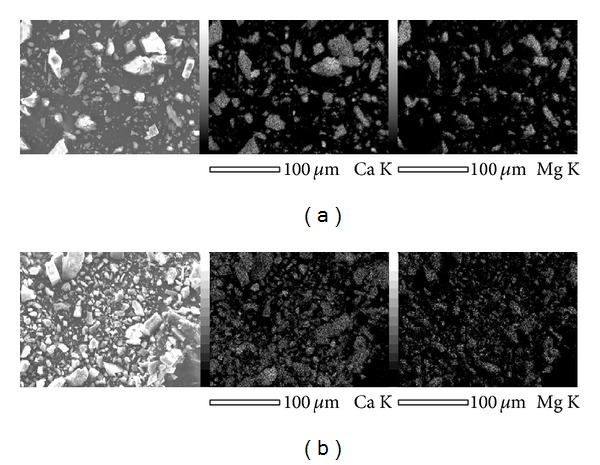
SEM micrographs and the energy-dispersive X-ray (EDX) analysis results of the dolomite pyrolysis products calcined in an Ar atmosphere (a) and vacuum (b).

**Figure 6:**
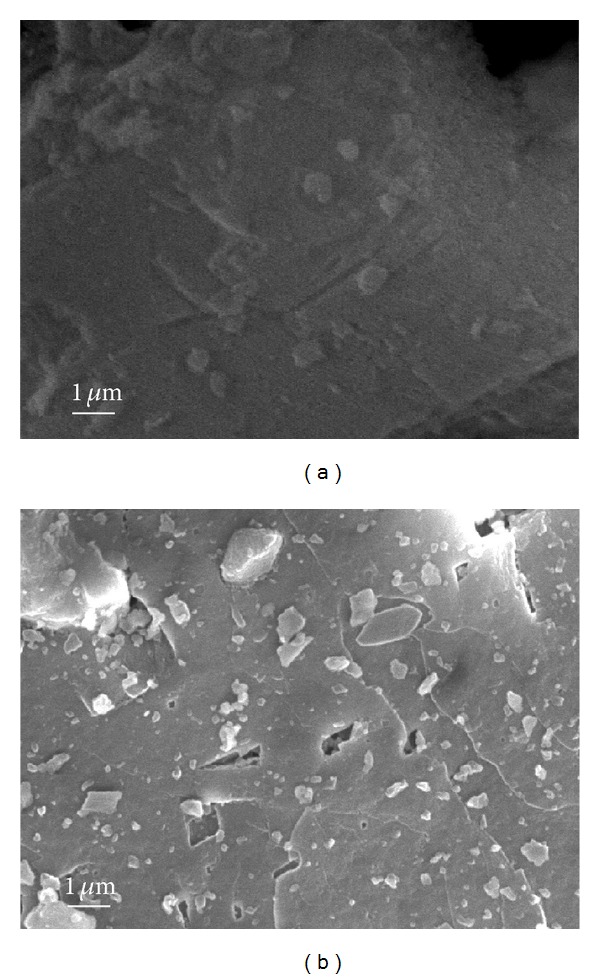
SEM micrographs of dolomite pyrolysis products ((a): Ar and (b): vacuum).

Figures 5 and 6 show the SEM images and the EDS analysis results of the dolomite samples. The surface morphologies of the calcined samples were different from those of the raw materials. The EDS analysis results indicate that there are CaO–MgO composite and CaO phase, being consistent with XRD analysis results.

### 3.3. Carbonation of Pyrolysis Products of Dolomite

**Figure 7:**
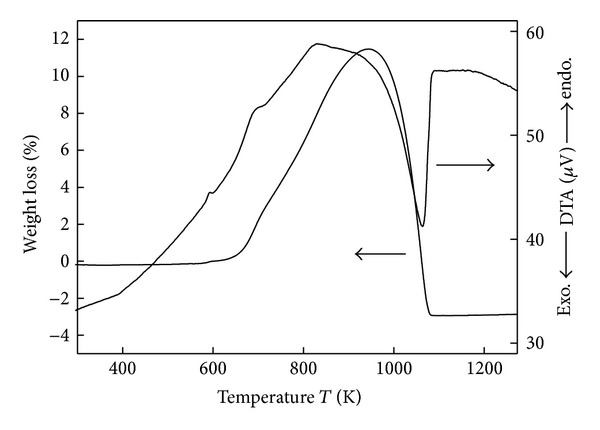
TG-DTA curves of dolomite pyrolysis products in CO_2_ atmosphere up to 1273 K.

TG-DTA curves of the pyrolysis products of dolomite heated to 1273 K in an atmosphere of CO_2_ are shown in Figure 7. The products were able to absorb CO_2_ between temperatures of 600 and 1100 K.  Figure 4 indicates that only CaO reacted with CO_2_ at these conditions. Previous studies [7, 18] also ratify this fact that MgO contained in calcined dolomite does not contribute to CO_2_ absorption. However, inert MgO stabilizes the structure of the solid sorbent during cyclic absorption and release procedures. Therefore, the stability of dolomite structure is attributed to the “excess” pore volume created by the original decomposition of MgCO_3_ in dolomite [7, 18].

**Figure 8:**
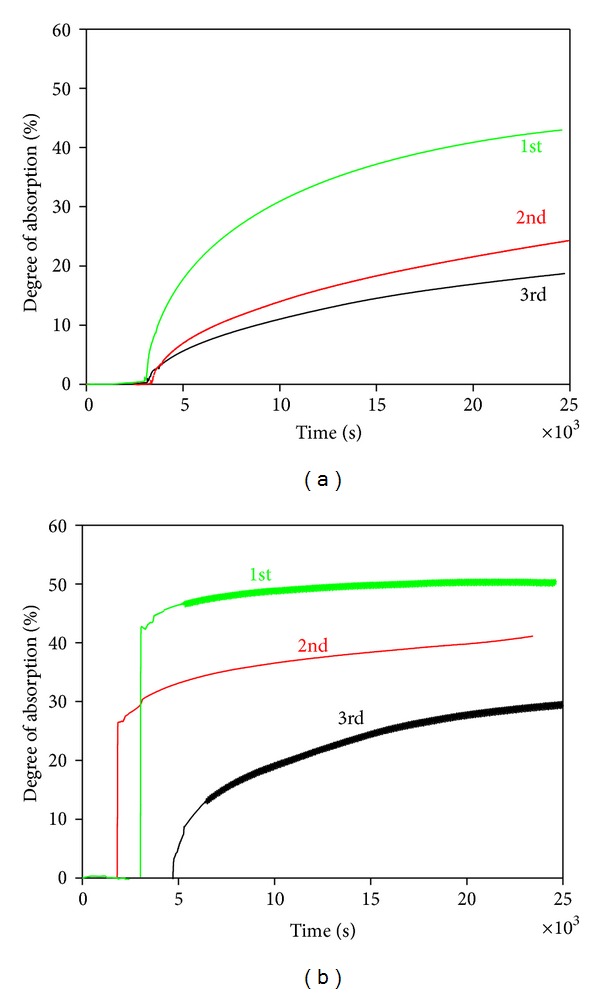
Cyclic degrees of absorption tests. (a): dolomite pyrolysis products calcined in Ar atmosphere; (b): calcined in vacuum.

Figure 8 shows the results of the cyclic degree of absorption tests. The degree of absorption of the dolomite samples calcined in an Ar atmosphere was 43% for the 1st cycle, 24% for the 2nd cycle, and 19% for the 3rd cycle. On the other hand, the degree of absorption of the dolomite sample calcined in vacuum was 51, 42, and 30% for the 1st, 2nd, and 3rd cycles, respectively. These values are clearly higher than those of the dolomite sample calcined in the Ar atmosphere because of the bigger specific surface area and higher micropore volume obtained.

**Figure 9:**
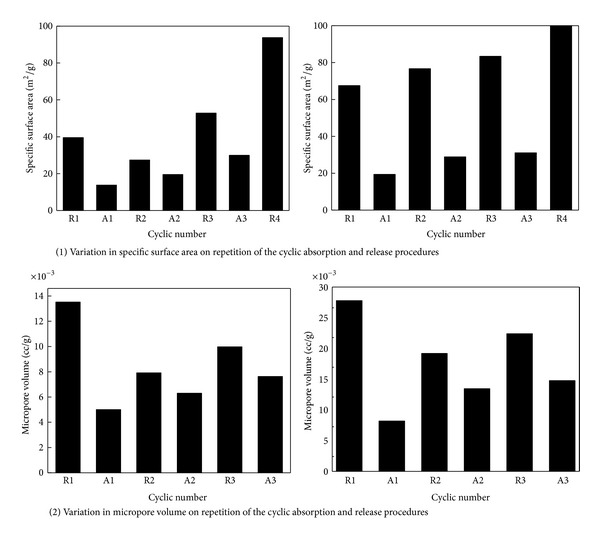
Variation of the specific surface area and micropore volume of calcined dolomite samples on cyclic absorption and release procedures. Dolomite samples calcined in the Ar atmosphere and in vacuum (b) (R: after CO_2_ release; A: after CO_2_ absorption).

Figure 9 shows variation of the specific surface area and micropore volume of calcined dolomite samples on cyclic absorption and release procedures. As can be seen, the higher absorption capacity of the dolomite sample calcined in vacuum is due to its high specific surface area and pore volume. Regardless of the calcination conditions used, the degree of absorption of the pyrolysis products of dolomite decreased with repeated cycling of the CO_2_ absorption and release procedures. Though, the specific surface area of CO_2_ released samples (*R*_*n*_) increased under repeated cycling, the pore volume did not increase. It is surmised that the difference between the expansion coefficients of MgO (10.5 × 10^−6^), CaO (13.6 × 10^−6^), and CaCO_3_ (11.7 × 10^−6^) leads to the pulverization of the dolomite particles. On the other hand, this fact also suggests that no new pores are formed in the CO_2_ release process.

**Figure 10:**
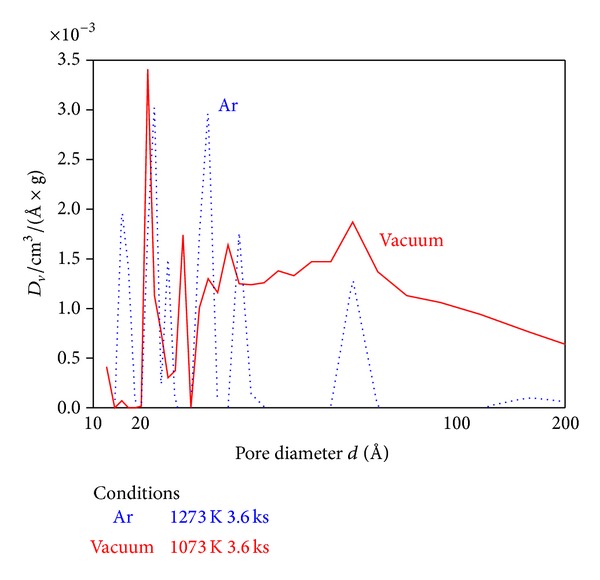
Pore diameter distribution of dolomite pyrolysis products.

Figure 10 shows distributions of the micropore diameter of calcined dolomite fabricated in the Ar atmosphere and vacuum. This figure indicates that dolomite pyrolysis product fabricated in vacuum has micropores with larger diameter.

### 3.4. Discussion

The calcination of dolomite leads to the formation of the mixture of CaO and MgO, which distribute uniformly in the pyrolysis product (see Figure 5). TG-DTA analysis revealed that only CaO can absorb CO_2_, while MgO is not active for CO_2_. These results indicate that the absorption of CO_2_ proceeds via the intraparticle diffusion process, wherein CO_2_ gas diffuses through the micropores in the CaCO_3_ and MgO mixture layer. On the absorption of CO_2_, the CaO phase expands leading to the blockage of CO_2_ diffusion paths, resulting in reduction of the specific reaction interface area (see Figure 8). Therefore, to fabricate CO_2_ absorbents of high capacity, not only a large specific surface area but also large pore volume and pore diameters are required.

Calcination in vacuum can lead to absorbents having large specific surface area, pore volume, and pore diameter, because of the reduction of reaction temperature causing the dolomite particles to aggregate less.

## 4. Conclusion

The CO_2_ absorption and release properties of the pyrolysis products of dolomite were evaluated using thermogravimetry. Dolomite was decomposed completely on calcination at 1273 K in an Ar atmosphere and at 1073 K in vacuum into particles of CaO and MgO. On subsequent absorption and release of CO_2_ in these particles, only CaCO_3_ was detected in the products along with unreacted CaO and MgO. The degree of absorption of CO_2_ was higher for dolomite calcined in vacuum than that calcined in the Ar atmosphere. Furthermore, the degree of absorption of CO_2_ of the calcined particles of dolomite decreased with successive cycles of CO_2_ absorption and release, because of the reduction of the size of the micropores in the particles.

## Figures and Tables

**Table 1:** Chemical composition of dolomite (wt%).

CaO	MgO	SiO_2_	Al_2_O_3_	Fe_2_O_3_	Others	Loss of fusion
34.53	17.92	0.29	0.08	0.04	0.76	46.38
